# Acetaldehyde Induces an Endothelium-Dependent Relaxation of Superior Mesenteric Artery: Potential Role in Postprandial Hyperemia

**DOI:** 10.3389/fphys.2019.01315

**Published:** 2019-10-22

**Authors:** Lexiao Jin, Pawel Lorkiewicz, Marina V. Malovichko, Aruni Bhatnagar, Sanjay Srivastava, Daniel J. Conklin

**Affiliations:** ^1^Department of Anesthesiology, Critical Care and Pain Medicine, The Second Affiliated Hospital and Yuying Children’s Hospital, Wenzhou Medical University, Wenzhou, China; ^2^Department of Pharmacology and Toxicology, University of Louisville, Louisville, KY, United States; ^3^Envirome Institute, University of Louisville, Louisville, KY, United States; ^4^Diabetes and Obesity Center, University of Louisville, Louisville, KY, United States; ^5^Department of Medicine, University of Louisville, Louisville, KY, United States; ^6^American Heart Association-Tobacco Regulation Center, University of Louisville, Louisville, KY, United States

**Keywords:** acetate, aldehyde dehydrogenase, EDRF, ethanol, feeding, mesenteric blood flow, nitric oxide

## Abstract

Acetaldehyde (AA) is a small, ubiquitous compound present in foods, beverages, as a gas phase combustion product, and also endogenously generated from metabolism as from ethanol (EtOH). Acetate is a short chain fatty acid derived from AA oxidation, and acetate levels were significantly higher in urine collected overnight with food provided *ad libitum* compared with urine collected after 9 h fasting. Feeding increases gastrointestinal blood flow, and thus, we explored the direct effects of AA (and acetate) in isolated murine superior mesenteric artery (SMA). Over the concentration range of 1–100 mM, AA strongly, and reversibly relaxed agonist-induced contractions of SMA including phenylephrine (PE), thromboxane A_2_ analog (U46,619) and high potassium (High K^+^) without toxicity. The sensitivity (EC_50_) but not the efficacy (>90% relaxation of PE-precontraction) of AA-induced relaxations was dependent on blood vessel (SMA was 3× more sensitive than aorta) and contractile agonist (PE EC_50_ = 3.3 ± 0.4 mM; U46,619 EC_50_ = 14.9 ± 1.5 mM; and High K^+^ EC_50_ = 17.7 ± 0.5 mM) yet independent of circadian cycle and sex. The most sensitive component of the AA-induced relaxation was inhibited significantly by: (1) a mechanically impaired endothelium; (2) nitric oxide synthase (NOS) inhibitor (L-NAME); and (3) a guanylyl cyclase (GC) inhibitor (ODQ). Both acetate and EtOH stimulated much weaker relaxations in SMA than did AA, yet these relaxations were significantly inhibited by L-NAME as well. Neither EtOH nor acetate relaxed pre-contracted aorta. Although neither cyanamide, a non-specific aldehyde dehydrogenase (ALDH) enzyme inhibitor, nor Alda-1, a specific activator of ALDH2 activity, had any effect on either sensitivity or efficacy of AA-induced relaxation in SMA, cyanamide significantly blocked both EtOH- and acetate-induced relaxations in SMA implicating a role of ALDH activity in vasorelaxation. These data show that AA relaxes SMA via an endothelium- and NO-dependent mechanism indicating that AA may be one component of the complex post-prandial hyperemia reflex via vasodilatation of mesenteric vasculature.

## Introduction

Acetaldehyde (AA) is the second smallest aldehyde, and its concentration in the blood is typically low due to high levels of aldehyde dehydrogenase (ALDH) activity throughout the body. AA is present in food and beverages and is from ethanol (EtOH) metabolism, which can be a major exogenous source of systemic AA, and AA may also enter via inhalation of combustion-derived aerosols (e.g., burning tobacco and vehicle exhaust) (Conklin et al., 2011; Ogunwale et al., 2017). Typically, AA levels range in the blood from 1 to 100 μM, and can reach >1 mM with excessive alcohol ingestion especially in individuals with an aldehyde dehydrogenase 2, *ALDH2^∗^2*, gene mutation, e.g., common in SE Asians (Tsukamoto et al., 1991; Vasiliou and Nebert, 2005). Blood levels of AA are constantly changing due to metabolism and exposures (e.g., feeding and inhalation) (Hobara et al., 1985), and thus, what physiological role AA plays in vascular control has remained uncertain.

A variety of direct effects of exogenous AA on cardiovascular targets have been described, yet a distinct role of AA in cardiovascular physiology is missing. For example, intra-arterial or intravenous AA reduces heart rate (bradycardia), decreases arterial pressure (hypotension) and causes arrhythmias in animals, but these are considered toxicological effects due to exceedingly high doses administered directly into the blood (Egle et al., 1973; Green and Egle, 1983). Nonetheless, AA is ubiquitous, and, it is thought that AA generated from EtOH contributes to alcohol-induced cardiomyopathy (Ren and Brown, 2000; Tan et al., 2012). Recently, it was shown that perfusion of a low level of AA (50 μM) into an isolated rat heart induces a cardioprotective effect against ischemia-reperfusion injury indicating that endogenous AA levels may be bioactive (Ueta et al., 2018). It is well-known that AA induces concentration-dependent relaxation in many different isolated blood vessels (Altura and Altura, 1982, 1987), yet the physiological relevance of AA-induced relaxation is uncertain. Yet, AA is considered relevant to the well-known phenomenon of EtOH-induced “flushing” (increased blood flow and redness in facial skin flushing) in people with an *ALDH2^∗^2* mutation that lowers ALDH2 activity, and thus, limits the rate of metabolism of AA to acetate (Scarino et al., 2009).

Despite myriad sources and well-known cardiovascular effects of AA, few studies have addressed the physiological vascular role of AA. To address this gap in knowledge, we measured urinary acetate levels, a primary metabolite of AA, as a potential biomarker of fluctuations in endogenous AA levels in mice. Urine levels of acetate after feeding overnight increased ≈2–3 times compared with urine levels after a daytime fast, and thus, it appeared that feeding increased endogenous AA levels (directly or indirectly). The gastrointestinal vasculature, thus, represents a likely target of AA levels after feeding when gastrointestinal blood flow increases reflexively (Takagi et al., 1988; Chou and Coatney, 1994). To test this idea, superior mesenteric artery (SMA) and aorta were isolated and then AA was added at physiological to supra-physiological concentrations (1–100 mM). The most sensitive AA-induced relaxation was in the SMA (1–30 mM), and it was dependent on: a functional endothelium, NO formation, and guanylyl cyclase activation. Because EtOH is metabolized to AA by alcohol dehydrogenases, and AA is metabolized by ALDH to acetate, the vascular effects of EtOH and acetate in the absence and presence of an ALDH enzyme inhibitor (cyanamide) and an ALDH2 activator (Alda1) were also assessed. EtOH and acetate (up to 30 mM) elicited less robust relaxations (<50% of AA-induced relaxation), yet these relaxations were NO- and ALDH-dependent in SMA indicating intrinsic overlap with AA’s mechanism of action. For many reasons, tobacco and EtOH are often consumed together (Abreu-Villaca et al., 2017), so the effect of nicotine on AA-induced relaxation also was tested. Collectively, these findings support the idea that increased levels of AA after feeding (and drinking of EtOH) likely contribute to feeding-associated increases in gastrointestinal blood flow, a reflex-driven process known as “postprandial hyperemia,” via sensitive, robust, reversible, endothelium-, and NO-dependent mechanisms.

## Materials and Methods

### Chemicals and Solutions

Reagent grade chemicals were purchased from Sigma-Aldrich (or as indicated): acetaldehyde (AA); acetate; acetylcholine chloride (ACh); Alda1 and cyanamide (gifts of Dr. B.G. Hill, University of Louisville); A967079 (AdooQ); 1h-[1,2,4]oxadiazolo[4,3-a]quinoxalin-1-one (ODQ); ethanol (100%); N^ω^-nitro-L-arginine methyl ester hydrochloride (L-NAME); nicotine bitartrate; L-phenylephrine hydrochloride (PE); sodium nitroprusside (SNP); U46,619 (thromboxane A_2_ analog); and 2,3,4,5-pentafluorobenzyl bromide (PFBBr).

Aortic Krebs PSS for aorta was (in mM): NaCl 118, KCl 4.7, CaCl_2_ 2.5, KH_2_PO_4_ 1.2, MgSO_4_ 1.2, NaHCO_3_ 12.5, and glucose 5.5; pH 7.4. SMA Krebs physiological salt solution (PSS) was (in mM): NaCl, 119; KCl, 4.7; MgCl_2_, 1.2; KH_2_PO_4_, 1.2; NaHCO_3_, 24; glucose, 7.0; pH 7.4. High potassium (60 mM) PSS (High K^+^) was prepared by substituting K^+^ equimolar for Na^+^.

### Animals

Wild type C57BL/6J mice (12–20 weeks old; 25–35 g) used in these studies were purchased (The Jackson Laboratory, Bar Harbor, ME, United States) or from in house breeding pairs. Aldose reductase (AR-null) null mice were from a breeding colony at University of Louisville (Wetzelberger et al., 2010). Mice were treated according to American Physiological Society *Guiding Principles in the Care and Use of Animals*, and all protocols were approved by University of Louisville IACUC. Mice were housed under pathogen-free conditions in a University of Louisville vivarium, i.e., controlled humidity and temperature, 12 h light: 12 h dark cycle. Mice were provided a standard chow diet (Rodent Diet 5010, 4.5% fat by weight, LabDiet; St. Louis, MO, United States).

### Urine Collection and Acetate Measurement

Urine samples were collected, centrifuged (200 × *g*, 10 min, 4°C), decanted, and stored at −80°C until analysis (Conklin et al., 2017). Each mouse was held (<5 s) and the mouth was gently touched with the sipper tube of a water bottle with D-glucose/saccharin solution (w/v; 3.0%/0.125% in water) immediately prior to a 6 h fast. After a 6 h fast, a single mouse was placed in each metabolic cage (Harvard Apparatus) with *ab libitum* glucose/saccharin solution without food for 3 h to collect fasting urine (4°C water-jacketed organ baths). Thus, mice were fasted for 9 h during daytime. After fasted urine collection, mice had food and glucose/saccharin solution *ab libitum* in overnight urine collection (O/N). Urine volumes were recorded, and samples were centrifuged to sediment food/feces, decanted, and stored at −80°C until analysis (Conklin et al., 2017).

As acetate is the primary urinary metabolite of AA, it was measured by GC-MS in electron ionization (EI) mode as described previously (Kage et al., 2004; Lamarre et al., 2014) with modifications (Conklin et al., 2018). Briefly, urine (50 μL) was mixed with sodium phosphate (20 μL 0.5M, pH 8.0) containing an internal standard (^13^C_1_D_3_-acetate, 2.3 mM). Samples were incubated with PFBBr (130 μL, 0.1M) for 15 min (60°C), extracted with hexane (330 μL), and analyzed by GC-MS in EI mode. A seven-point calibration curve was used to calculate the concentration of acetate, and acetate level was normalized to urinary creatinine (mg) to account for urine dilution (Lorkiewicz et al., 2018).

### Isolated Aorta and SMA

The aorta and SMA were removed via mid-ventral thoracotomy from anesthetized mice (sodium pentobarbital, 0.1 ml, 150 mg/kg, i.p.). Thoracic aorta rings (3–4 mm) were hung on stainless steel hooks in 15-ml water-jacketed organ baths, and SMA rings (2 mm) were hung on tungsten wire (75 μm dia.) in 5-ml heated organ baths (MultiWire Myograph System 620M, DMT, Denmark) in PSS bubbled with 95% O_2_:5% CO_2_ (37°C). After 10 min without tension, aorta rings were equilibrated to ≈1 g tension (30 min), and SMA rings were equilibrated to ≈0.25 g tension (1 h). All rings were stimulated with High K^+^ to test for viability, washed 3-times with PSS (30 min), and re-equilibrated to designated tension (Jin et al., 2019).

### AA-Induced Relaxation in Isolated Aorta and SMA

To study relaxation, acetaldehyde (AA; 1–100 mM) was added to aorta and SMA pre-contracted with 3 different contractile agonists: phenylephrine (PE, 10 μM); U46,619 (0.1 μM); and High K^+^ (60 mM). The efficacy of AA-induced relaxation was calculated as the maximal% reduction in agonist-induced contraction (E_max_). The sensitivity of agonist-induced relaxation was assessed as the effective concentration producing 50% response (EC_50_), i.e., cumulative concentration responses were normalized to 100% with EC_50_ interpolation (Conklin et al., 2009). To see whether time of day affected SMA response to AA, male mice were euthanized both in daytime and in nighttime (Jin et al., 2019).

### Role of AA Metabolism and Effects of EtOH and Acetate (AA Metabolite)

Because EtOH is a known source of AA, we tested whether EtOH alone (up to 1%, equivalent to amount present in 100 mM AA) mimicked AA-induced relaxation. Because AA oxidation by ALDH leads to acetate formation, we tested whether acetate (1–100 mM) also induced vasorelaxation in PE pre-contracted aorta, and SMA. To test the specific role of ALDH activity to AA-induced vasorelaxation, we used a non-specific ALDH enzyme inhibitor (cyanamide, 1 mM) and an activator of ALDH2 (Alda-1, 25 μM) (Srivastava et al., 2001; Chen et al., 2008). In addition, to test for a role of AA reduction, SMA and aorta were isolated from aldose reductase (AR) null mice.

### Endothelium, Nitric Oxide Synthase (NOS), and cGMP Mechanisms

To assess the contribution of the endothelium to vasorelaxation, the endothelium was mechanically injured by air perfusion, and effective impairment was confirmed by >95% loss of ACh-induced relaxation of PE pre-contracted blood vessels (Jin et al., 2019). The contribution of NOS to relaxation was tested by addingL-NAME (0.1 mM) prior (15 min) to PE (10 μM), U46,619 (0.1 μM), or High K^+^ (60 mM). As NO increases guanylyl cyclase (GC) activity and formation of cGMP, blood vessels were pre-incubated with ODQ (3 μM) to inhibit GC (Jiang et al., 2015) to test for a role of cGMP in AA-induced relaxation.

### Transient Receptor Potential Ankyrin 1 (TRPA1)

Because formaldehyde (smallest aldehyde) induces a sensitive TRPA1-dependent relaxation in isolated murine SMA (Jin et al., 2019), we tested whether the TRPA1 antagonist (A967079, 3 μM) also would alter AA-induced relaxation in PE-precontracted SMA.

### Effects of Nicotine and AA

In tobacco users who drink EtOH, which is common, elevated exposures to AA and nicotine occur simultaneously, and thus, to test for any interaction between these compounds, nicotine (1 μM) was applied before cumulative addition of AA (1–100 mM) in PE pre-contracted SMA.

### Statistical Analyses

Data are expressed as means ± SE. For comparing two groups, a paired or unpaired *t*-test was used (as appropriate). Multiple group testing was done with Kruskal-Wallis ANOVA and Bonferroni *post hoc* test. Statistical significance was accepted at *P* < 0.05.

## Results

### Acetate Primary Metabolite of Acetaldehyde (AA): Effect of Feeding

Acetate is a major oxidation metabolite of AA, and thus, urinary acetate is an indirect measure of AA metabolism. Thus, we measured urinary acetate by GC-MS, and we found that *ad libitum* feeding during night time significantly increased urinary levels of acetate compared with fasting levels collected during daytime cycle. In both female and male C57BL/6J mice, feeding overnight increased urinary acetate levels (normalized to creatinine) by 2–3× over daytime fasting levels (Table 1). Although acetate is a well-known short chain fatty acid product of gut microbiota metabolism, we infer that feeding likely increased AA levels, and that AA was subsequently metabolized by ALDH to acetate, which then was excreted in urine.

**TABLE 1:** Urinary acetate levels of either a daytime fast or overnight feeding in **(A)** female and **(B)** male C57BL/6J mice.

	**Condition**	
**Urinary metabolite**	**Daytime fast**	**Overnight feeding**
**A. Female mice**		
Acetate (μg/ml)	2.76 ± 0.27	24.52 ± 4.89^∗^
Creatinine (mg/dl)	3.19 ± 0.41	8.74 ± 1.52
Acetate (μg/mg creatinine)	91.11 ± 6.61	296.82 ± 32.95^∗^
**B. Male mice**		
Acetate (μg/ml)	4.71 ± 0.66	8.54 ± 2.34^∗^
Creatinine (mg/dl)	6.52 ± 1.16	5.67 ± 1.15
Acetate (μg/mg creatinine)	79.56 ± 7.49	158.57 ± 32.16^∗^

*Values = means ± SE (*n* = 11–12 mice/group). ^∗^*P* < 0.05 vs. Daytime fast.*

### AA-Induced Relaxation

**FIGURE 1:**
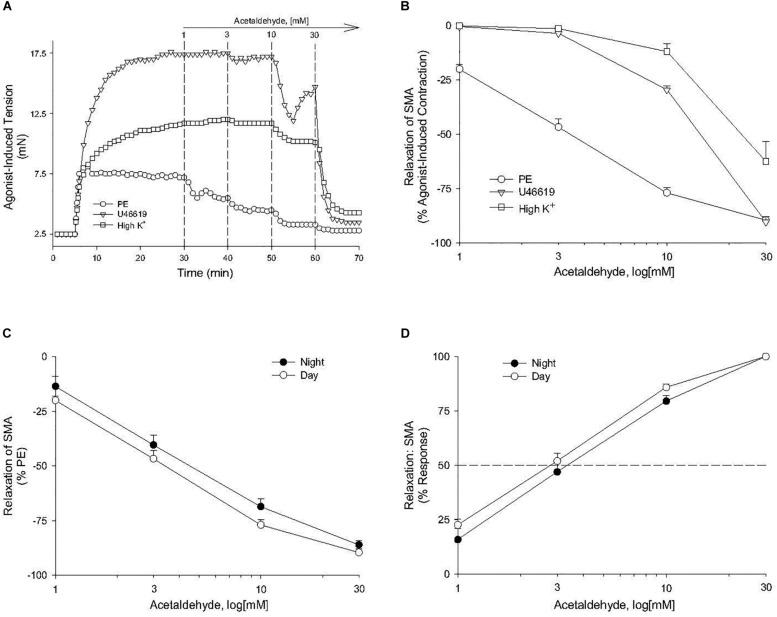
Acetaldehyde (AA) induced relaxation in superior mesenteric artery (SMA). **(A)** Representative traces of concentration-dependent relaxation stimulated by cumulative addition of AA (1–100 mM) in isolated SMA pre-contracted by one of 3 agonists: phenylephrine (PE); thromboxane A_2_ analog (U46,619); or high potassium (High K^+^). **(B)** Summary graph of the efficacy (E_max_: % relaxation) of AA-induced relaxation in isolated PE, U46, 619, and High K^+^ pre-contracted SMA. **(C)** Summary graph of the efficacy (E_max_) and of the **(D)** sensitivity (EC_50_) of AA-induced relaxation in isolated PE-precontracted SMA from male mice euthanized either during day time or night time. Values are means ± SE of SMA (*n* = 3–4 mice).

Because feeding increases gastrointestinal (GI) tract blood flow, we investigated whether AA would relax superior mesenteric artery (SMA) – a major conduit for increased GI blood flow with feeding (Chou and Coatney, 1994; Lucchini et al., 1996). AA (1 to 100 mM) did induce a robust, repeatable, and concentration- and agonist-dependent relaxation in precontracted SMA and aorta of female and male mice (Figure 1 and Table 2). Regardless of precontraction agonist (PE, U46,619, and High K^+^), AA relaxed tension by >90% in both SMA (Figures 1A,B) and in aorta (Table 2; PE only). Yet when AA was added to an uncontracted blood vessel, it did not affect basal tone (i.e., myogenic tone; data not shown). Blood vessel sensitivity of male SMA was circadian-independent, and sensitivity of female SMA in daytime was different from male SMA sensitivity (day cycle) (Figures 1C,D and Table 2), and thus, subsequent experiments to probe the mechanisms of AA-induced relaxation were conducted using only SMA isolated from male mice in the daytime (lights on). Collectively, AA-induced relaxation could be repeated (up to 100 mM) without loss of efficacy or any change in ACh-induced, endothelium-dependent relaxation indicating little toxicity of AA even at supra-physiological levels (data not shown).

**TABLE 2:** The sensitivity of acetaldehyde-induced relaxation (effective concentration inducing 50% relaxation, EC_50_; in mM) in isolated murine superior mesenteric artery (SMA, female and male mice) and thoracic aorta (male only) with different contractile agonists.

**Blood vessel (sex and time of day)**	**EC_50_ by pre-contraction agonist**		
	**PE**	**U46,619**	**High K^+^**
SMA (f, day)	6.0 ± 0.6^∗^	–	–
SMA (m, day)	3.3 ± 0.4	14.9 ± 1.5^∗^	17.7 ± 0.5^∗^
SMA (m, day) + L-NAME	6.2 ± 0.2^∗^	17.2 ± 0.2	18.4 ± 0.4
SMA (m, night)	4.3 ± 0.8	–	–
Aorta (m)	15.9 ± 3.0^∗^	–	–

*Values = means ± SE (*n* = 3–10 mice/group). PE, phenylephrine 10 μM; U46,619, thromboxane A_2_ analog 0.1 μM; High K^+^, high potassium solution; –, did not perform experiment. ^∗^*P* < 0.05 vs. EC_50_ of “SMA (male, day)” PE agonist (i.e., baseline control).*

### Endothelium, NOS, and GC Mechanisms in AA-Induced Relaxation

**FIGURE 2:**
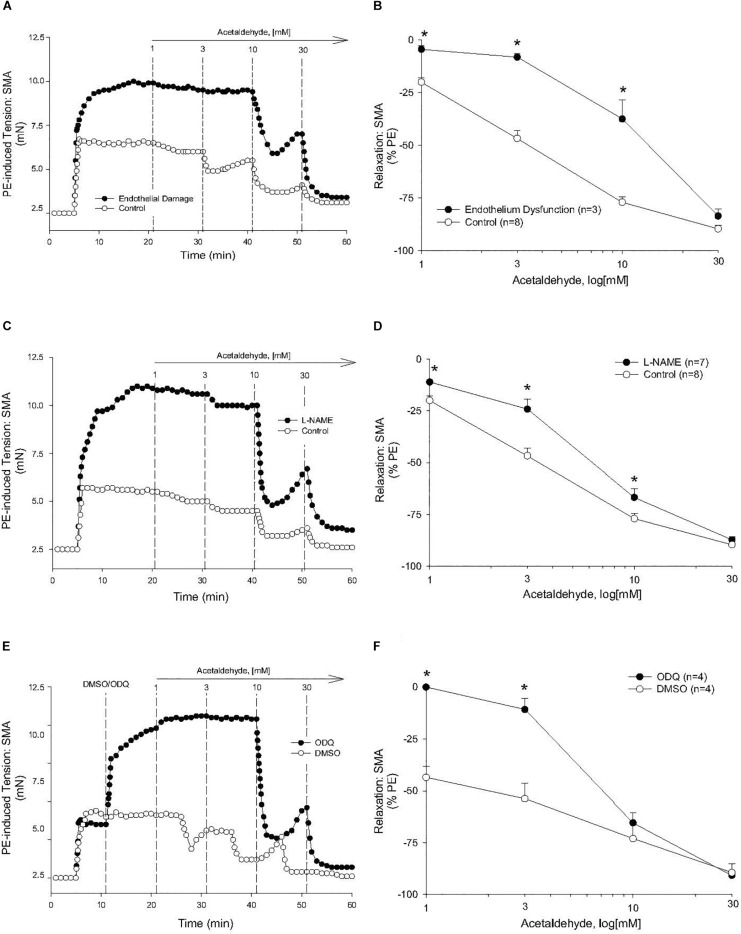
Endothelium, nitric oxide (NO) and guanylyl cyclase (GC) mechanisms in acetaldehyde-induced (AA) relaxation of SMA. **(A,B)** Representative traces and summary data of AA-stimulated relaxation of PE-induced contraction in SMA with intact endothelium or with impaired endothelium (15 min of air perfusion). **(C,D)** Representative traces and summary data of AA-stimulated relaxation of PE-induced contraction in the absence of and presence of NOS inhibitor, L-NAME. **(E,F)** Representative traces and summary graph of AA-induced relaxation of PE-induced contraction in the absence of and presence of the GC antagonist (ODQ, 3 μM). ODQ was added to bath after PE-induced contraction stabilized yet prior to cumulative addition of AA. Values are means ± SE of SMA (*n* = 3–8 mice). ^∗^*P* < 0.05 vs. Control.

The sensitivity of AA-induced relaxation (but not efficacy) was agonist-dependent (PE < U46,619 = High K^+^; Figures 1A,B and Table 2) and indicated at least 2 distinct mechanisms were operative. We showed the most sensitive component of the AA-induced relaxation was significantly rightward-shifted by mechanical disruption of endothelium in SMA (air perfusion impaired >90% of ACh-induced relaxation) (Figures 2A,B and Tables 3, 4). This effect was similar to the effect of L-NAME incubation, which further implicated endothelium-derived NO (Figures 2C,D and Tables 3, 4). Because NO activates guanylyl cyclase (GC) and cGMP formation in smooth muscle cells, we used ODQ as an irreversible inhibitor of GC. ODQ significantly rightward-shifted the AA-induced relaxation (Figures 2E,F and Tables 3, 4). Collectively, these data show that the most sensitive component of the AA-induced relaxation was dependent on a functional endothelium, NOS (likely eNOS) and an NO-mediated activation of GC, but at high concentrations, the relaxation of AA was unaffected (Table 3). For example, L-NAME shifted the sensitivity of AA-induced relaxation in PE pre-contracted SMA (3.3 ± 0.3 vs. 6.3 ± 0.6 mM), but did not alter efficacy (E_max_). Similarly, L-NAME neither affected the sensitivity nor efficacy of AA-induced relaxation SMA pre-contracted with U46,619 (17.2 ± 0.3 vs. 14.9 ± 1.5 mM) or High K^+^ (18.4 ± 0.4 vs. 17.7 ± 0.5 mM) (Supplementary Figures S1A,B and Table 2). These data demonstrate two components of AA-induced relaxation: a sensitive endothelium-derived NO component and an insensitive and NO-independent mechanism.

**TABLE 3:** Efficacy (maximal relaxation, E_max_) of acetaldehyde- (AA), EtOH- and acetate-induced relaxations of PE-precontracted superior mesenteric artery (SMA), and aorta without and with treatments.

**Compound**	**mM**		**Treatment**	**E_max_ (% relaxation)**	
	**SMA**	**Aorta**		**SMA**	**Aorta**
AA	1–30	1–100	Control	89.6 ± 1.7	91.6 ± 6.7
AA	1–30	–	ED	83.5 ± 3.4	–
AA	1–30	–	+L-NAME	87.1 ± 4.0	–
AA	1–30	–	+ODQ	90.8 ± 1.8	–
AA	1–30	–	+A967079	90.6 ± 4.0	–
AA	1–30	–	+nicotine	83.8 ± 3.1	–
EtOH	1–30	1–100	Control	39.4 ± 3.1	0
EtOH	1–30	–	+L-NAME	8.1 ± 1.2^∗^	–
Acetate	1–30	1–100	Control	42.9 ± 5.9	0
Acetate	1–30	–	+L-NAME	16.0 ± 8.2^∗^	–

*Values = means ± SE (*n* = 3–6 mice/group). PE, phenylephrine 10 μM; ED, endothelial dysfunction induced by mechanical air perfusion; L-NAME, N^ω^-nitro-L-arginine methyl ester hydrochloride 100 μM; A967079, TRPA1 antagonist 1 μM; ODQ, 1h-[1,2,4]oxadiazolo[4,3-a]quinoxalin-1-one 3 μM; –, did not perform experiment. ^∗^*P* < 0.05 vs. untreated and matched Control.*

**TABLE 4:** The sensitivity (effective concentration inducing 50% relaxation, EC_50_; in mM) of acetaldehyde- (AA), EtOH- and acetate-induced relaxations in isolated murine superior mesenteric artery (SMA, male mice only) pre-contracted with PE in the absence and presence of functional endothelium, inhibitors, or nicotine.

**Compound**	**Control EC_50_**	**Treatment**	**Treatment EC_50_**
**(1–30 mM)**	**(vehicle)**		
AA	3.9 ± 0.6	+ED	12.4 ± 2.1^∗^
AA	2.9 ± 0.4	+L-NAME	6.2 ± 0.6^∗^
AA	2.1 ± 0.9	+ODQ	7.4 ± 0.9^∗^
AA	2.9 ± 0.6	+A967079	2.8 ± 0.6
AA	5.4 ± 1.4	+nicotine	3.6 ± 1.6
EtOH	3.2 ± 1.5	+L-NAME	X
acetate	6.9 ± 3.0	+L-NAME	X

*Values = means ± SE (*n* = 3–6 mice/group). PE, phenylephrine 10 μM; ED, endothelium dysfunction induced by mechanical air perfusion; L-NAME, N^ω^-nitro-L-arginine methyl ester hydrochloride 100 μM; ODQ, 1h-[1,2,4]oxadiazolo[4,3-a]quinoxalin-1-one 3 μM; A967079, TRPA1 antagonist 3 μM; nicotine, 1 μM; X, could not calculate EC_50_ due to low % relaxation (see Table 3). ^∗^*P* < 0.05 vs. Control (vehicle).*

### Ethanol-, AA-, and Acetate-Induced Relaxations

**FIGURE 3:**
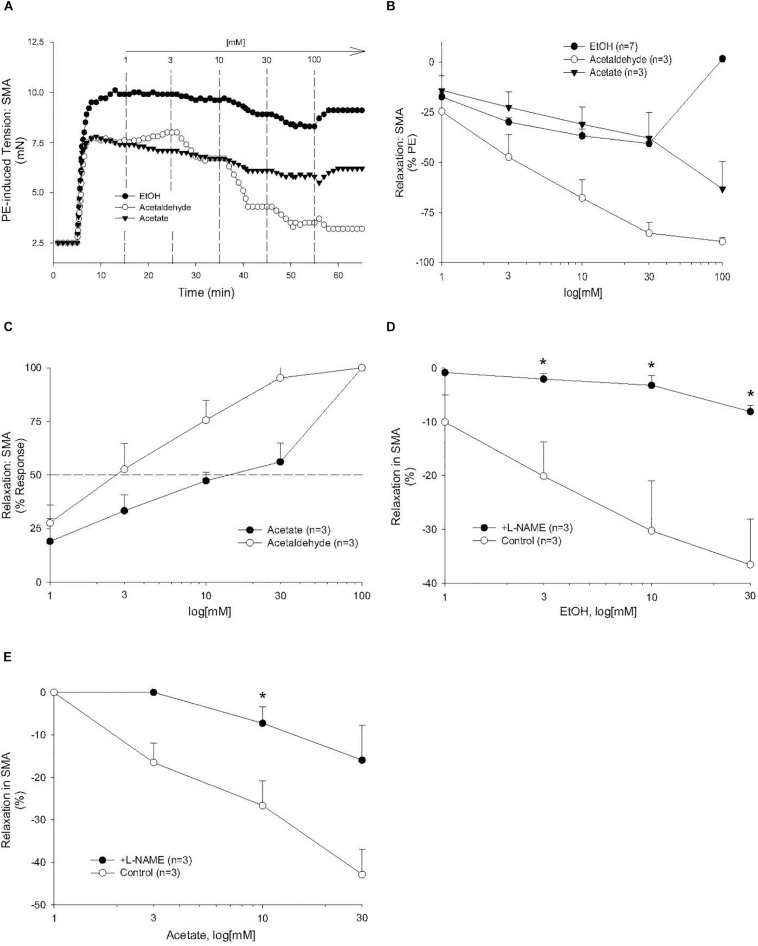
The vascular effects of ethanol (EtOH), AA, and acetate in SMA. Role of the nitric oxide (NO) in ethanol- and acetate-induced relaxations of SMA. **(A)** Representative traces of EtOH-, AA-, and acetate-induced relaxations in PE pre-contracted SMA. Summary data of EtOH-, AA-, and acetate-induced relaxations in PE pre-contracted SMA plotted as measures of efficacy (**B**, % PE contraction), and sensitivity (**C**, converted to 100% response). Concentration dependent relaxations induced by either **(D)** ethanol or **(E)** acetate of phenylephrine (PE) pre-contracted superior mesenteric artery (SMA) in the absence and presence of L-NAME. Values are means ± SE of SMA (*n* = 3 mice). ^∗^*P* < 0.05 vs. Control.

Because AA is formed from EtOH by alcohol dehydrogenases (ADH), and AA is metabolized by ALDH to acetate, it is possible that the vascular effects of EtOH or acetate are due to formation of AA. To address this possibility, EtOH or acetate was cumulatively added (up to 100 mM) in PE pre-contracted SMA and aorta. Ethanol is known to induce vasodilation in several blood vessels, yet it is unclear if the effect is due to formation of AA. Ethanol relaxed PE-precontracted SMA weakly (E_max_ about 40% of PE precontraction) compared with AA (Figures 3A,B). Ethanol did not relax PE pre-contracted aorta even at 100 mM, in fact, it increased tension primarily at 100 mM (Supplementary Figure S2A) in contrast to AA relaxation (Supplementary Figures S2B,C). Acetate also induced weak relaxation with a slight contraction at 100 mM in PE-precontracted SMA (Figures 3A–C). L-NAME treatment significantly reduced both EtOH- and acetate-induced relaxations of SMA (Figures 3D,E and Table 3). Although both EtOH- and acetate-induced significantly weaker relaxations in SMA than did AA, the NO dependence of these relaxations was a shared mechanism of action with AA.

### Role of AA Metabolism: Effects of Cyanamide and Alda1 and AR

**FIGURE 4:**
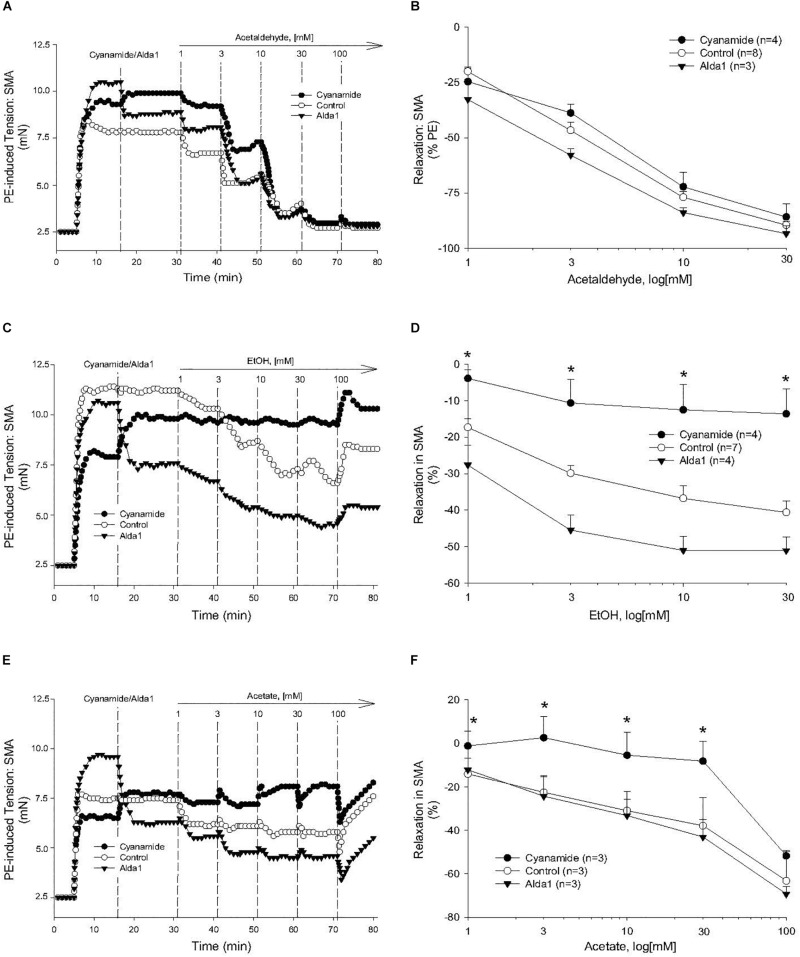
Role of aldehyde dehydrogenase (ALDH) metabolism in vascular responses to EtOH, AA, and acetate. Representative traces and summary data, respectively, of vasorelaxant effects of AA **(A,B)**, EtOH **(C,D)**, and acetate **(E,F)** in PE pre-contracted SMA of WT mice in the absence and presence of either cyanamide (ALDH enzyme inhibitor, 1 mM) or Alda1 (ALDH2 activator, 25 μM). Values are means ± SE of SMA (*n* = 3–8 mice). ^∗^*P* < 0.05 vs. Control.

**FIGURE 5:**
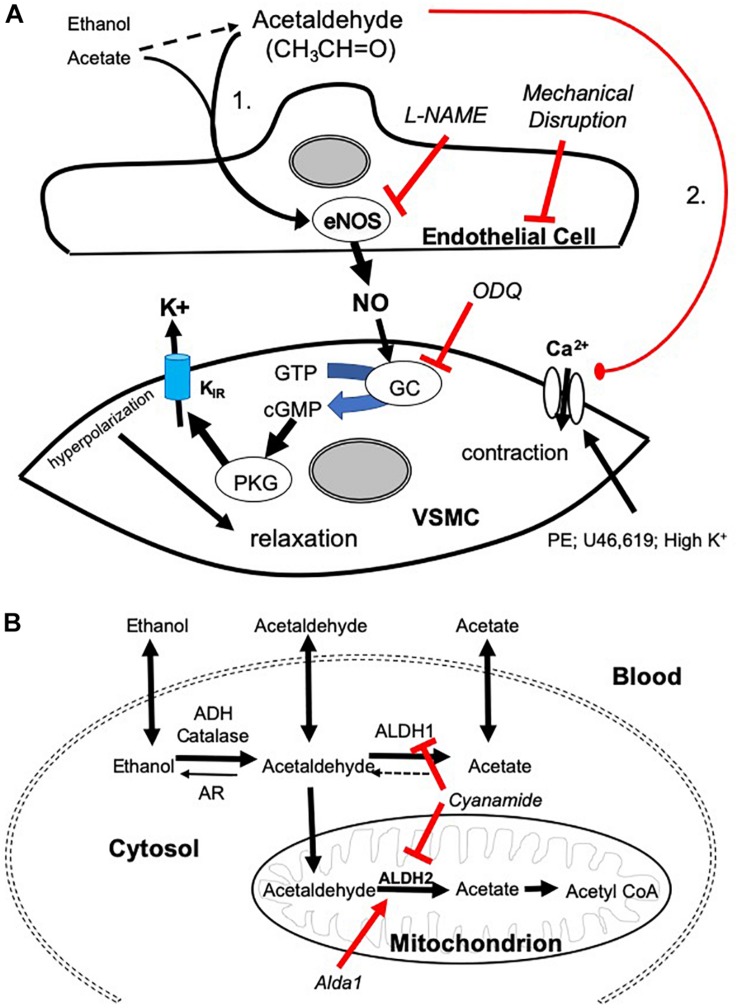
Mechanisms of AA-induced vasorelaxation and relationship to aldehyde metabolism. **(A)** Cartoon showing mechanisms by which AA induces relaxation in SMA pre-contracted with phenylephrine (PE), thromboxane A_2_ analog (U46,619), and high potassium solution (High K^+^). AA stimulates a sensitive endothelial cell-dependent pathway that leads to eNOS activation, NO formation, and GC dependence triggering VSMC hyperpolarization and relaxation (Pathway is designated “1”). Several steps were confirmed by antagonizing endothelium (impaired by air perfusion), NOS (L-NAME), and GC (ODQ). A less sensitive, yet equally efficacious, pathway of relaxation included a voltage-insensitive mechanism that similarly reversed U46,619- and High K^+^-induced contraction implicating closure of VSMC Ca^2+^ channels (Pathway “2”). **(B)** Cartoon highlighting the metabolic pathways of ethanol, AA, and acetate. Ethanol is oxidized to AA by alcohol dehydrogenases (ADH) and/or catalase activity. AA is oxidized to acetate by aldehyde dehydrogenases (ALDH/ALDH2) or reduced back to EtOH by aldose reductase (AR). Acetate may be converted to acetyl coenzyme A (CoA).

To investigate the possible role of metabolism in AA-induced responses, cyanamide and Alda-1, ALDH enzyme inhibitor and an ALDH2 activator, respectively, and an aldose reductase (AR) null mouse model were used. Surprisingly, and despite vasoactive effects of their own, cyanamide (increased tension, 10–20%) and Alda-1 (decreased tension, 10–20%), neither compound had any significant effect on AA-induced relaxation in PE-precontracted SMA (Figures 4A,B and Table 5). However, the sensitivity of the AA-induced relaxation of SMA was rightward shifted significantly in AR-null mice (EC_50_, 6.9 ± 1.0 mM, *n* = 3) although no effect on maximal relaxation was observed (E_max_, 78.1 ± 4.0%). Despite lack of a strong effect of ALDH modulators on AA’s vasoactivity, cyanamide significantly blocked the relaxation of EtOH, and Alda1 slightly enhanced the EtOH-induced relaxation (Figures 4C,D and Table 5). Similarly, cyanamide significantly blocked the relaxation of acetate, although Alda1 was without effect on acetate-induced relaxation (Figures 4E,F and Table 5). These data support a likely role of ALDH activity in EtOH- and acetate-induced relaxations in SMA (see Figure 5).

**TABLE 5:** Role of aldehyde dehydrogenase (ALDH) in AA-induced relaxations of PE-precontracted SMA.

**SMA**	**E_max_ (% Relaxation of PE-precontraction)**		
**Compound (1–30 mM)**	**Control (vehicle)**	**+Cyanamide**	**+Alda1**
**A. Efficacy**			
Acetaldehyde	89.5 ± 4.8	85.9 ± 5.9	93.4 ± 3.5
Ethanol	40.6 ± 3.1	13.6 ± 6.8^∗^	52.2 ± 4.4
Acetate	38.1 ± 5.1	8.2 ± 9.1^∗^	43.1 ± 8.0

**SMA**	**EC_50_, [in mM]**		

**Compound (1–30 mM)**	**Control (vehicle)**	**+Cyanamide**	**+Alda1**

**B. Sensitivity**			
Acetaldehyde	2.5 ± 0.4	3.7 ± 0.7	2.0 ± 0.3
Ethanol	1.5 ± 0.2	X	1.2 ± 0.4
Acetate	2.4 ± 0.8	X	3.6 ± 0.9

*The efficacy (A; measured as maximal relaxation, E_max_;%) and sensitivity (B; measured as effective concentration inducing 50% relaxation, EC_50_; in mM) of AA-, EtOH-, and acetate-induced relaxations in isolated murine superior mesenteric artery (SMA; male) pre-contracted with PE in the presence of cyanamide (ALDH inhibitor) or Alda1 (ALDH2 activator). Values = means ± SE (*n* = 3–6 mice/group). PE, phenylephrine 10 μM; X, could not calculate EC_50_ due to low % relaxation; Cyanamide, 1 mM; Alda1, 25 μM. ^∗^*P* < 0.05 vs. Vehicle Control.*

### Role of Transient Receptor Potential Ankyrin 1 (TRPA1)

Although formaldehyde induces a sensitive TRPA1- and endothelium-dependent relaxation in PE-precontracted SMA, AA-induced relaxation in PE-precontracted SMA was unaffected by addition of the TRPA1 antagonist (A967079, 1 μM) (Tables 3, 4) indicating this pathway is not activated in a similar way by AA.

### Nicotine and AA

In tobacco users, EtOH, and thus, AA, may be present in blood simultaneously with nicotine, so we also tested whether nicotine altered AA-induced relaxation in SMA. Nicotine alone (1 μM) had no effect in PE-contracted SMA, and also had no effect on subsequent AA-induced vasorelaxation (Tables 3, 4).

## Discussion

Although the physiological role of AA’s vascular action is still uncertain, our study provides evidence for a potential contribution of AA in postprandial hyperemia in SMA via an EDRF-mediated dilatory pathway. In support of this hypothesis, we show that SMA is significantly more sensitive than the aorta is to AA. This is important because the SMA is a major arterial blood supply to the gastrointestinal tract and it dilates after feeding due to a cadre of factors, including nutrients, vago-vagal reflex, NO, substance P, etc. (Chou and Coatney, 1994; Lucchini et al., 1996). Moreover, we observed circadian fluctuation in urinary acetate levels indicating that endogenous AA level likely increases after feeding (see Table 1). Because AA is naturally abundant in foods, water and beverages (Committees on Aldehydes, 1981; Conklin et al., 2011) including as major metabolic by product of EtOH, AA is a plausible stimulus for increasing gut blood flow after feeding. As this is the first suggestion of this specific physiological role of AA, certainly more research is necessary to establish AA as a *bona fide* stimulus of postprandial hyperemia *in vivo*.

We established two distinct pathways of AA-induced relaxation in pre-contracted SMA (Figure 5A), and we focused on mechanisms of the most sensitive pathway as this is more likely a physiological pathway. Consistent with this supposition, the sensitive pathway of AA-induced relaxation requires: a functional endothelium, NOS activity (presumably eNOS), and guanylyl cyclase activity (GC/cGMP). The endothelial cells likely contribute to the most sensitive components, whereas the latter component is likely present in vascular smooth muscle cells (VSMC). Interestingly, these components also are activated by formaldehyde in both rat aorta and murine SMA (Zhang et al., 2018; Jin et al., 2019). Despite the similarity, AA did not activate the TRPA1 channel as did formaldehyde indicating AA triggers a different “activating step” in the endothelium. L-NAME, an eNOS inhibitor, significantly blocks the most sensitive component of the AA-induced relaxation and shifts the EC_50_ to the right. Yet this shift is less strong than that of mechanical endothelium disruption or ODQ treatment (GC inhibitor) suggesting that a second endothelium-dependent component likely is operative. We did not rule out that other components of the vascular wall may contribute to the sensitive pathway of AA-induced relaxation in SMA (e.g., perivascular nerves, etc.) (Nilius et al., 2012).

Acetaldehyde induced a much stronger relaxation in SMA (and aorta) than either an upstream precursor, EtOH, or its downstream metabolite, acetate – both of which stimulate vasorelaxation in SMA but not in aorta indicating important similarities and differences with AA. EtOH-induced relaxations are well-documented (half century), yet the full mechanism of this phenomenon remains incomplete. EtOH induces vascular relaxation by acting on both endothelial cells and VSMCs to dilate mesenteric and cutaneous blood vessels; yet EtOH also contracts cerebral, coronary, pulmonary, and renal vascular beds and skeletal muscle arterioles (Altura and Altura, 1982, 1987; Altura et al., 1983; Toda et al., 1983; Greenberg et al., 1993; Ru et al., 2008; Rocha et al., 2012). Our data show that both EtOH and acetate relax SMA at concentrations <100 mM whereas 100 mM induces contraction. In contrast, EtOH and acetate only increase tension in pre-contracted aorta. Nonetheless, L-NAME significantly blocks the most sensitive component of both EtOH- and acetate-induced relaxation in SMA reflecting NO-dependence (endothelium) – as is observed for AA (see shared Pathway 1 in Figure 5A). Because relaxations in SMA induced by EtOH or acetate are qualitatively similar to AA, this may reflect a role of AA in both EtOH- and acetate-induced relaxations. As AA induces relaxation in both SMA and aorta this likely reflects a common yet significantly less sensitive pathway (#2; see Figure 5A) in both blood vessels stimulated by extracellular AA (but not by EtOH or acetate).

The concentration of AA in human blood is kept very low due to fast acting aldehyde dehydrogenase 2 (ALDH2) enzyme. The capacity of the liver to eliminate AA formed from EtOH is so efficient that measurable levels of AA are typically undetected in peripheral blood (Lindros et al., 1980), but AA is detected in saliva. In an alcohol drinking model, salivary AA concentration was approximately 150 μM (maximum 260 μM) over the first 20 to 40 min followed by a rapid decrease to 20 to 30 μM for the remainder of the follow-up period (350 min) (Salaspuro, 2017). Moreover, ALDH2 enzyme deficiency is present in 35–45% of Asian people (8% of world’s population), and results from *ALDH2^∗^2* gene mutation that decreases ALDH activity by 20% (heterozygous) and 50% (homozygous), and thus, people with *ALDH2^∗^2* gene mutation are susceptible to facial flushing and nausea after alcohol consumption presumably due to high AA levels (Chen et al., 2014; Gross et al., 2015). Consistent with epidemiological findings, alcohol drinking in ALDH2-deficient subjects markedly elevates AA concentrations in saliva (Vakevainen et al., 2000; Yokoyama et al., 2008), and in blood of EtOH- or AA-injected ALDH2-deficient mice (Jamal et al., 2016). Interestingly, high levels of AA are associated with increased risk of GI tract cancers (Salaspuro, 2017) indicating that AA is likely elevated in the GI tract as well.

Although concentrations of salivary and blood AA in humans and those used in our study are difficult to compare, theoretically, consuming as little as one glass of wine (5 oz or 150 ml) containing 15–20 g of EtOH (11–13% ABV) would increase blood EtOH concentration to 65 mM (assuming 5 l blood volume, 70 kg adult). As EtOH is rapidly metabolized to AA by ADH in liver and in blood vessels, it is plausible that AA levels increase quite quickly in endothelial cells and VSMC with corresponding intracellular/membrane levels being likely in the bioactive range as described in our study (1–30 mM). Despite limits of theoretical calculation, there is little evidence for circulating AA levels in the active dilation range presumably because of rapid intracellular oxidative metabolism of AA to acetate by ALDH activity (see Figure 5B). To test the role of SMA-dependent ALDH metabolism on AA’s bioactivity, we use an ALDH inhibitor, cyanamide, and an ALDH2 activator, alda-1, to decrease and increase ALDH activity, respectively, and in turn, potentially augment and diminish AA’s action. Interestingly, Alda-1 (25 μM) and cyanamide (1 mM) are vasoactive in SMA with opposing effects: Alda-1 relaxed; cyanamide contracted (see Figure 4E). Because of these opposing effects, we infer that the pharmacological target (ALDH2 enzyme activity) may be an active regulator of SMA vascular tone. Perhaps more surprising, neither compound significantly alters AA-induced relaxation, yet cyanamide effectively blocks both EtOH- and acetate-induced relaxations in SMA. In contrast to our hypothesis, wherein we expect cyanamide (or AR-null) to enhance EtOH-induced relaxation by increasing AA level, we observe inhibition of EtOH-induced relaxation. Notably, this result is not without precedent. For example, EtOH combined with cyanamide inhibits rather than enhances EtOH-induced increases in blood flow (Carmichael et al., 1987). Interestingly, AA alone did not increase portal blood flow either. Equally provocative is our finding that Alda-1 increases the relaxation of EtOH but does not alter relaxation of either AA or acetate. Collectively, these data are both compelling (ALDH *activity likely is involved in vascular effects of EtOH and acetate*.) and vexing (*Why are vascular effects of AA unaltered by modulators of* ALDH *activity?*). The latter may be a function of extracellular addition of AA whereas both EtOH and acetate need to be intracellular in order to be metabolized to bioactive AA. The cyanamide-mediated inhibition of EtOH- and acetate-induced relaxations further indicates a dependence on ALDH activity for initiation of these relaxations. There is, of course, precedent for such a role of ALDH2 activity in nitroglycerin-induced vasorelaxation (Hink et al., 2007; Beretta et al., 2012), however, others show that cyanamide alone induces relaxation in isolated, precontracted rabbit aorta (Fukuto et al., 1994). Moreover, as nicotine did not alter AA-induced SMA relaxation, we infer nicotine does not inhibit vascular ALDH activity. To dissect out the complex biochemical interplay between AA vascular action and ALDH activity will require further studies.

## Conclusion

In conclusion, we elucidate the mechanism of a sensitive component of AA-induced relaxation in SMA that is sequentially dependent on EDRF/NO and GC/cGMP pathways. We suggest this relaxation is a likely contributor to a broader, multifaceted postprandial hyperemic reflex that enhances blood flow to the GI tract to aid digestion and nutrient absorption. Moreover, EtOH and acetate (a short chain fatty acid metabolite of gut microbiota) may contribute to local vascular AA levels (via ADH and ALDH mediated metabolism) that bolster the reflex increase in gut blood flow related to feeding (and drinking).

## Data Availability Statement

The datasets analyzed in this manuscript are not publicly available. Requests to access the datasets should be directed to the corresponding author.

## Ethics Statement

The animal study was reviewed and approved by the Institutional Animal Care and Use Committee, University of Louisville, Louisville, KY, United States.

## Author Contributions

LJ and DC planned, conducted the experiments, generated, analyzed and interpreted data, and wrote the manuscript. PL and MM generated and interpreted data. AB and SS helped with the analysis, interpretation of the data, and contributed to the editing of the manuscript. DC was the guarantor of this work and had full access to all data and took responsibility for the integrity and accuracy of the data.

## Conflict of Interest

The authors declare that the research was conducted in the absence of any commercial or financial relationships that could be construed as a potential conflict of interest.

## Supplementary Material

FIGURE S1 Role of the nitric oxide (NO) in AA-induced relaxations of SMA. AA (1–100 mM) stimulated relaxation of isolated SMA pre-contracted by one of 3 agonists: phenylephrine (PE); thromboxane A_2_ analog (U46,619); or high potassium (High K^+^). Summary graphs of the **(A)** efficacy (% relaxation) and of the **(B)** sensitivity of AA-induced relaxation in isolated PE-precontracted SMA in the absence and presence of L-NAME. Values are means ± SE of SMA (*n* = 3–8 mice).

FIGURE S2 The vascular effects of EtOH, AA, and acetate in aorta. **(A)** Representative traces of EtOH-, AA-, and acetate-induced responses in PE pre-contracted aorta. Summary graphs of AA-induced relaxations in PE pre-contracted aorta plotted as measures of efficacy (**B**, % PE contraction), and sensitivity (**C**, converted to 100% response). Values are means ± SE (*n* = 5 mice).
