# Supplementary figures and images for: Acetaldehyde Induces an Endothelium-Dependent Relaxation of Superior Mesenteric Artery: Potential Role in Postprandial Hyperemia
Source: Front Physiol. 2019 Oct 22;10:1315. doi: 10.3389/fphys.2019.01315 (PMC6817488; doi:10.3389/fphys.2019.01315)

# Suppl. Fig. 1

A.

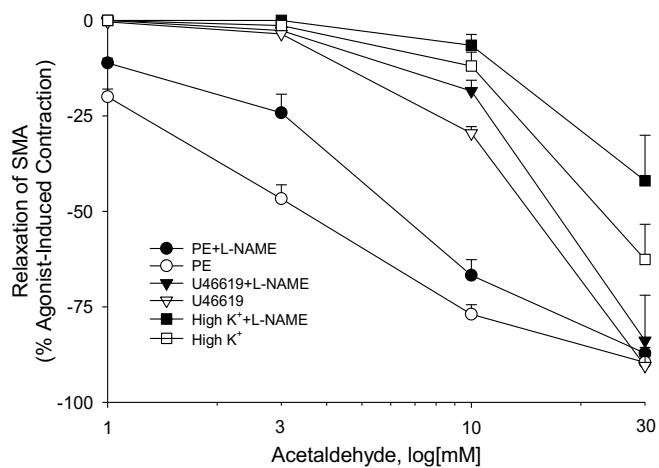

B.

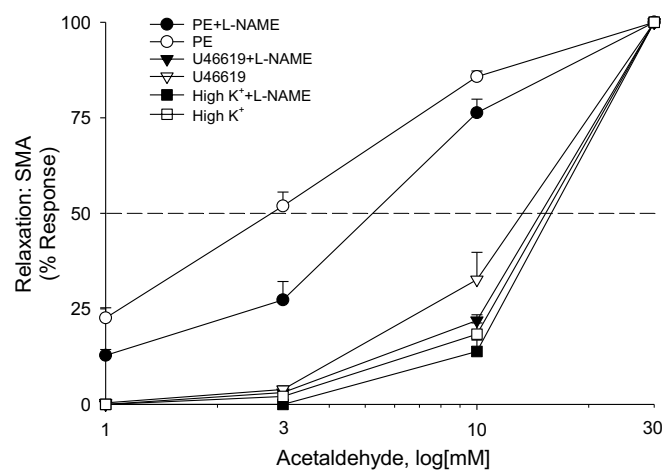

Supplement: FIGURE S1 — Role of the nitric oxide (NO) in AA-induced relaxations of SMA. AA (1–100 mM) stimulated relaxation of isolated SMA pre-contracted by one of 3 agonists: phenylephrine (PE); thromboxane A2 analog (U46,619); or high potassium (High K+). Summary graphs of the (A) efficacy (% relaxation) and of the (B) sensitivity of AA-induced relaxation in isolated PE-precontracted SMA in the absence and presence of L-NAME. Values are means ± SE of SMA (n = 3–8 mice). [file Data_Sheet_1.PDF]

# Suppl. Fig. 2

A.

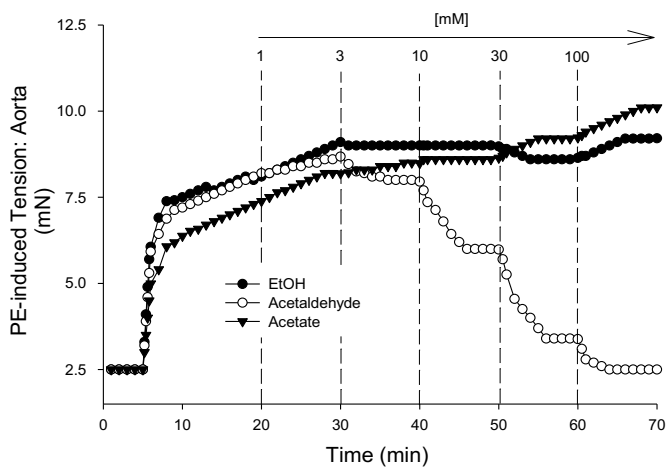

B.

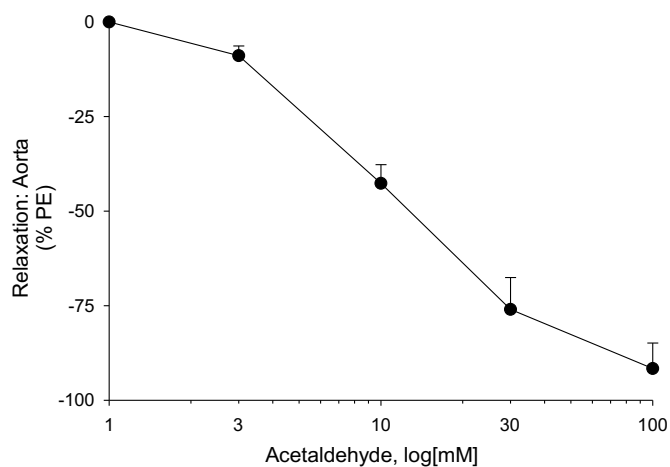

C.

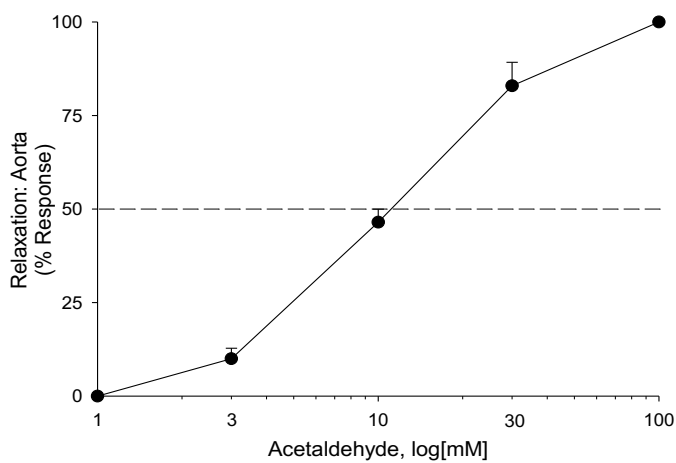

Supplement: FIGURE S2 — The vascular effects of EtOH, AA, and acetate in aorta. (A) Representative traces of EtOH-, AA-, and acetate-induced responses in PE pre-contracted aorta. Summary graphs of AA-induced relaxations in PE pre-contracted aorta plotted as measures of efficacy (B, % PE contraction), and sensitivity (C, converted to 100% response). Values are means ± SE (n = 5 mice). [file Data_Sheet_2.PDF]
